# MicroRNA210 Suppresses Mitochondrial Metabolism and Promotes Microglial Activation in Neonatal Hypoxic–Ischemic Brain Injury

**DOI:** 10.3390/cells14151202

**Published:** 2025-08-05

**Authors:** Shirley Hu, Yanelly Lopez-Robles, Guofang Shen, Elena Liu, Lubo Zhang, Qingyi Ma

**Affiliations:** 1 The Lawrence D. Longo Center for Perinatal Biology, Department of Basic Sciences, School of Medicine, Loma Linda University, Loma Linda, CA 92350, USA; 2 Department of Hematologic Malignancies Translational Science, Beckman Research Institute of City of Hope, Duarte, CA 91010, USA

**Keywords:** neuroinflammation, mitochondrial dysfunction, microglia, hypoxia–ischemia

## Abstract

Neuroinflammation is the major contributor to the pathology of neonatal hypoxic–ischemic (HI) brain injury. Our previous studies have demonstrated that microRNA210 (miR210) inhibition with antisense locked nucleic acid (LNA) inhibitor mitigates neuroinflammation and provides neuroprotection after neonatal HI insult. However, the underlying mechanisms remain elusive. In the present study, using miR210 knockout (KO) mice and microglial cultures, we tested the hypothesis that miR210 promotes microglial activation and neuroinflammation through suppressing mitochondrial function in microglia after HI. Neonatal HI brain injury was conducted on postnatal day 9 (P9) wild-type (WT) and miR210 knockout (KO) mouse pups. We found that miR210 KO significantly reduced brain infarct size at 48 h and improved long-term locomotor functions assessed by an open field test three weeks after HI. Moreover, miR210 KO mice exhibited reduced IL1β levels, microglia activation and immune cell infiltration after HI. In addition, in vitro studies of microglia exposed to oxygen–glucose deprivation (OGD) revealed that miR210 inhibition with LNA reduced OGD-induced expression of *Il1b* and rescued OGD-mediated downregulation of mitochondrial iron–sulfur cluster assembly enzyme (ISCU) and mitochondrial oxidative phosphorylation activity. To validate the link between miR210 and microglia activation, isolated primary murine microglia were transfected with miR210 mimic or negative control. The results showed that miR210 mimic downregulated the expression of mitochondrial ISCU protein abundance and induced the expression of proinflammatory cytokines similar to the effect observed with *ISCU* silencing RNA. In summary, our results suggest that miR210 is a key regulator of microglial proinflammatory activation through reprogramming mitochondrial function in neonatal HI brain injury.

## 1. Introduction

Neonatal hypoxic–ischemic encephalopathy (HIE) is a leading cause of acute mortality and long-term disability in newborns [1,2,3,4,5]. Neuroinflammation is the key contributor to HI injury in the neonatal brain [6,7,8]. After the onset of HIE, the immune response takes place within hours and lasts several days to weeks depending on the severity of the insult [9,10]. The overproduced proinflammatory cytokines and chemokines attract the migration and infiltration of peripheral immune cells such as monocytes and neutrophils through the compromised blood–brain barrier (BBB) to the sites of brain injury [11,12,13]. As a result, the inflammation is enhanced, which ultimately triggers secondary neuronal cell death and delayed brain damage after HI insult [11,12,14,15,16]. As the primary brain-resident immune cells, microglia are one of the earliest responders to insults and engage in the onset and progression of HI pathophysiological changes [12,17,18,19]. It has been documented that the state of microglial activation may reflect the severity of brain injury post-stroke [12,19]. However, the acute activation of microglia under hypoxia–ischemia conditions remains elusive.

Mitochondrial metabolic reprogramming drives microglia–macrophage proinflammatory activation [20,21]. When activated, microglia are subjected to a metabolic shift from primarily using oxidative phosphorylation (OXPHOS) toward increased glycolysis to generate energy, which is crucial for their rapid response to various conditions such as inflammation in CNS disorders [20,22]. The glycolytic shift in microglia–macrophage is linked to the overproduction of proinflammatory cytokines, contributing to the progression of neuroinflammation [21,23]. The molecular regulation of metabolic reprogramming in microglia is largely unclear. We have demonstrated that microRNA210 (miR210) orchestrates neuroinflammation after hypoxia–ischemia brain injury in rodent models of neonates and adults [24,25]. Moreover, miR210 has been identified as a master regulator of mitochondrial bioenergetic metabolism in many cell types, such as neurons, macrophages, and endothelial cells, particularly during hypoxia [25,26,27]. This evidence suggests a novel mechanistic link between miR210 and microglial activation during HIE.

In the present study, we investigated the underlying mechanism of miR210 in the regulation of microglial proinflammatory activation after HIE. Using miR210 knockout mice, we found that miR210 deletion reduced the expression of proinflammatory cytokines, microglial activation and immune cell infiltration after HIE. Next, we tested the role of miR210 in mitochondrial metabolic changes and proinflammatory response using oxygen–glucose deprivation model in BV2 microglia or isolated primary murine microglia. Our results indicate that miR210 reprogramming mitochondrial bioenergetics promotes proinflammatory response in microglia after HI.

## 2. Methods

### 2.1. Animals

MiR210 heterozygous mice with a C57BL/6J genetic background were generously provided by Drs. Huang and Sadovsky [28]. The genotypes of the offspring were confirmed via PCR. In experiments, all the procedures and protocols were approved by the Institutional Animal Care and Use Committee of Loma Linda University and followed the guidelines of the National Institutes of Health Guide for the Care and Use of Laboratory Animals. A total of about 145 mouse pups were used in animal experiments, of which 115 were included in the final data analyses. The authors complied with the ARRIVE guidelines, including randomization, blinding, power calculation-based sample size estimation details of the animals, exclusion and inclusion criteria, outcome assessment, inclusion of control groups, and statistical methods. For brain infarct assay, pups without brain lesion after HI insult were excluded from both WT and KO groups. Mice were housed in plastic cages on a 12 h light/dark cycle with access to water ad libitum and a standard laboratory diet.

### 2.2. Neonatal Mouse Model of Hypoxia–Ischemia (HI)

A modified Rice–Vannucci model was produced on postnatal day 9 (P9) mouse pups, as described previously [29,30,31,32]. Briefly, mouse pups were fully anesthetized with inhalation of 2% isoflurane. The right common carotid artery (CCA) in the neck was exposed and ligated with an 8.0 silk surgical suture (Fine Science Tools, Foster City, CA, USA). After surgery, pups were returned to their dams for 1 h and then placed in a hypoxic incubator (Thermo Fisher Scientific, Riverside, CA, USA) containing 10% oxygen at 37 °C for 60 min. At the end of hypoxia, pups were returned to their dams for recovery. For the sham group, the right CCA of mouse pup was exposed but without ligation and hypoxia. Mouse pups of mixed males and females were randomly assigned to each experimental group.

### 2.3. Measurement of Brain Infarct Size

Brain infarct size was determined using 2,3,5-triphenyltetrazolium chloride monohydrate (TTC, Sigma-Aldrich, St. Louis, MO, USA) staining 48 h after HI as we previously described [25]. Briefly, the brain was isolated from each pup, dissected into coronal sections (2 mm thickness, 4 slices per brain), and immersed into pre-warmed 2% TTC in PBS (Sigma-Aldrich, St. Louis, MO, USA) for 10 min at 37 °C against light. Sections were washed with PBS and fixed by 10% formaldehyde (Fisher Scientific, Waltham, MA, USA) overnight. The caudal and the rostral surfaces of each slice were photographed using a digital camera, and the percentage of infarct area (average of both sides) in the ipsilateral hemisphere for each slice was traced and analyzed by the NIH Image J software 1.54e. The data were analyzed by an observer blinded to experimental groups.

### 2.4. Open Field Test

The open field test [33] was conducted three weeks after HI (one-month old) in a square test box (40 × 40 × 40 cm^3^). Each mouse was placed in the arena and allowed to freely explore the box for 10 min, which was recorded by an overhead camera (Logitech, San Jose, CA, USA). The distance moved and average speed were calculated by analyzing the recorded video. The data were analyzed via NIH ImageJ software 1.54e with the MouBeAT plugin [34] by an investigator blinded to experimental groups.

### 2.5. Cell Culture and Treatment

BV2 microglia were provided by Dr. Grace Y. Sun (University of Missouri). Cells were cultured in Dulbecco’s modified Eagle’s medium (DMEM; Hyclone Co., Logan, UT, USA) with 10% fetal bovine serum (FBS; Hyclone Co., Logan, UT, USA), 2 mM L-glutamine (Thermo Fisher Scientific, Riverside, CA, USA), 100 U/mL penicillin/streptomycin (Corning, Corning, NY, USA) in a humidified incubator (Eppendorf, San Diego, CA, USA) with 5% CO_2_ at 37 °C. miR210-LNA (100 nM) and LNA scramble control (Qiagen, Germantown, MD, USA) were used for in vitro transfection. Transfection was performed with Lipofectamine 2000 transfection reagent (Invitrogen, Carlsbad, CA, USA) according to the manufacturer’s instructions. BV2 microglia were starved in serum-free medium for 1 h before transfection. At 24 h after transfection, cells were treated with hypoxia (1% oxygen, 5% CO_2_/94% N_2_) at 37 °C for 3 h in glucose-free medium and collected for assays.

Primary microglia were isolated from the cerebral cortices of postnatal day 2–3 mouse pups, as described previously [35,36]. Briefly, the brains were collected in cold HBSS (Corning, Corning, NY, USA) and the meninges were removed. Cerebral cortices were dissociated in culture medium for glial cells (DMEM supplemented with 10% FBS, 1 mM l-glutamine, 1 mM sodium pyruvate (Thermo Fisher Scientific, Riverside, CA, USA), 100 U/mL penicillin/streptomycin). After passage through 70 µm filters (Corning, Corning, NY, USA), cells were seeded in poly-L-lysine-coated T-75 flasks (Corning, Corning, NY, USA) and placed in a humidified incubator with 5% CO_2_ at 37 °C. The medium was changed after 24 h. On days 14–21, microglia were shaken off the mixed glial cell cultures using an orbital shaker. Isolated primary microglia were seeded onto pre-coated plates or glass coverslips according to the assays, and grown in culture medium (ScienCell, Carlsbad, CA, USA) for microglia (RPMI-1640 supplemented with 10% FCS, 1 mM l-glutamine, 1 mM sodium pyruvate, 0.1 mM non-essential amino acids, 50 mM β-mercaptoethanol, 100 U/mL penicillin/streptomycin). Experiments were initiated 24 h after seeding, when cultures consist primarily of microglia. Microglia were treated with miR210 mimic (50 nM) (Qiagen, Germantown, MD, USA), Stealth *ISCU* silencing RNA (50 nM) (Thermo Fisher Scientific, Riverside, CA, USA) or their negative controls with Lipofectamine 2000. Medium was changed 4 h after transfection to reduce the toxicity of transfection reagent. At 48 h after transfection, cells were collected for assays.

### 2.6. Seahorse for Mito Stress Assay

The mitochondrial respiratory function was tested using the Mito Stress assay kit by a Seahorse XFe24 analyzer (Agilent, Santa Clara, CA, USA), according to the manufacturer’s instruction. Briefly, the cartridge plate was hydrated with calibrate buffer and incubated overnight (37 °C, CO_2_-free). The assay medium was prepared immediately before assay. The real-time changes in oxygen consumption rate (OCR) were measured after sequential injection of an ATP synthase inhibitor oligomycin (1.5 µM, OM), H^+^ ionophore (cyanide p-trifluoromethoxyphenyl-hydrazone (1.5 µM, FCCP), or electron transport chain inhibitors rotenone and antimycin A (0.5 µM, ROT/AA). The real-time extracellular acidification rate (ECAR) was detected using a modified protocol for the Mito Stress test, as previously reported [37]. Briefly, the assay medium was prepared with glutamine (2 mM). During the assay, the glucose (10 mM) was injected into the assay medium followed by sequential injection of OM, FCCP combined with sodium pyruvate (1 mM), and ROT/AA. Each treatment has at least three replicated wells. The readout per well was normalized to the total protein amount measured with the Pierce™ BCA Protein Assay Kit (Thermo Fisher Scientific, Riverside, CA, USA) at the end of assay.

### 2.7. Flow Cytometry

The contralateral and ipsilateral hemispheres of the mouse brain were collected after removing the cerebellum. The mononuclear cells were separated through the 70/30% percoll gradients (Fisher Scientific, Waltham, MA, USA), as we previously described [24]. Briefly, the hemisphere was mechanically dissociated to single-cell suspension in RPMI buffer and passed through a 70 µm nylon cell strainer. After centrifugation, cell pellet was resuspended in 7 mL 30% percoll and layered it on top of 70% percoll. The solution was then centrifuged with 500× *g* at room temperature for 30 min in the absence of a brake. The cells were collected from the interphase of two density gradients, incubated with anti-mouse CD16/CD32 antibody (BioLegend, San Diego, CA, USA) to block Fc fragment receptor (FcR), and stained with a mix of fluorochrome-conjugated antibodies for 20 min at 4 °C. The following antibodies were used, including Fixable viability dye eF506, CD45-Alexa Fluor^®^ 647, and CD11b-Alexa Fluor^®^ 488 (Bio-Rad, Hercules, CA, USA). After washing, cells were fixed in 2% paraformaldehyde (Sigma-Aldrich, St. Louis, MO, USA) and analyzed in an MACSQuant Analyzer 10 flow cytometer (Miltenyi Biotec, Gaithersburg, MD, USA). The multi-color compensation was performed using MACS Comp bead kit. The data were analyzed using Flowmagic and FlowJo v10 software. Cells were then gated for CD45+ populations. Microglia (CD45high/CD11b+) and lymphocytes (CD45+/CD11b−) were separated from the latter population [38].

### 2.8. Real-Time Quantitative PCR (RT-qPCR)

Total RNA was subjected to reverse transcription with the Superscript III First-Strand Synthesis System (Invitrogen, Carlsbad, CA, USA) according to the manufacturer’s instructions. The target gene mRNA abundance was determined with real-time PCR using iQ SYBR Green Supermix (Bio-Rad, Hercules, CA, USA). The following primers were used:

*Il1b* (F: GAGTGTGGATCCCAAGCAAT; R: TACCAGTTGGGGAACTCTGC), *Il6* (F: CCACGGCCTTCCCTACTTC; R: TGGGAGTGGTATCCTCTGTGAA), *Tnf-α* (F: CAGCCGATGGGTTGTACCTT; R: GGCAGCCTTGTCCCTTGA), *ISCU* (F: CCTGTGAAACTGCACTGCTC; R: TCTCTGGCTCCTCCTTCTTG), *Ndufa4* (F: TCCCAGCTTGATTCCTCTCTT; R: GGGTTGTTCTTTCTGTCCCAG), *Sdhd* (F: CGAAAGCGACATGGCGGTTC; R: GGTCCTGGAGAAATGCTGACAC), *Gapdh* (F: CGACAGTCAGCCGCATCTT; R: CCAATACGACCAAATCCGTTG). Real-time PCR was performed in a final volume of 25 μL, and each PCR reaction mixture consisted of specific primers and iQ SYBR Green Supermix.

MiR210 levels were analyzed by miScript II RT kit and miScript SYBR Green PCR kit with miScript Primer Assay kit (Qiagen, Germantown, MD, USA) according to the manufacturer’s instructions, as we previously described [25]. Briefly, 1 μg of template RNA was mixed with reverse-transcription master mix in a final volume of 20 μL and incubated at 37 °C for 60 min, and then the reaction was stopped at 95 °C. A total of 2 ng of template cDNA were used for miR210 quantification in a final volume of 25 μL system containing specific primers and QuantiTect SYBR Green PCR master mix according to the manufacturer’s instructions. Primers included miScript Universal Primer, miR210 miScript Primer Assay, and SNORD61 miScript Primer Assay (Qiagen, Germantown, MD, USA).

PCR was performed in triplicate, and threshold cycle numbers were averaged for each sample. The relative expression levels were calculated using the Formula 2^(−ΔΔCt)^ and normalized to actin. The change in mRNA abundance was expressed as a fold of normal control.

### 2.9. Enzyme-Linked Immunosorbent Assay (ELISA)

The levels of IL1β were quantified using ELISA Kit (R&D, Minneapolis, MN, USA) according to the manufacturer’s instructions, as we previously described [24]. Briefly, after lysis of the ipsilateral hemisphere of mouse brains, total protein concentration was determined by Pierce BCA protein assay. The absorbance was read with the TECAN microplate reader (TECAN, San Jose, CA, USA). All samples were analyzed in duplicates, and the data were normalized to the concentration of IL1β in total protein concentration.

### 2.10. Western Blotting

Protein extraction of cultured cells was obtained using cell lysis buffer (Cell Signaling, Danvers, MA, USA) with further centrifugation for 10 min at 14,000× *g* at 4 °C. The supernatant was collected, and the protein concentration was determined using the Pierce BCA protein assay kit. Equal amounts of protein were loaded on an SDS-PAGE gel. After being electrophoresed and transferred to a PVDF membrane (Bio-RAD, Hercules, CA, USA), the membrane was blocked and incubated with the primary antibody at 4 °C overnight. The primary antibodies included the following: rabbit anti-ISCU (Proteintech, Rosemont, IL, USA) and rabbit polyclonal anti-GAPDH antibody (Abcam, Cambridge, MA, USA). Then, membranes were incubated with secondary antibodies (Santa Cruz Biotechnology, Santa Cruz, CA, USA) at room temperature for 1 h. Immunoblots were then probed with an ECL Plus chemiluminescence reagent kit (Fisher Scientific, Waltham, MA, USA) and exposed to Hyperfilm (Fisher Scientific, Waltham, MA, USA). The images were analyzed by densitometry using the NIH Image J software 1.54e and normalized to GAPDH.

### 2.11. Statistical Analysis

Data were expressed as mean ± standard error of the mean (SEM). All graphs in this study were generated with GraphPad Prism 9.0.2. In experiments related to animals, experimental number (*n*) represents pups from at least two different dams. All in vitro experiments were performed at least in triplicates. Comparisons between two groups were analyzed using Student’s *t* test (unpaired, two-tailed), and multiple comparisons were analyzed using one-way ANOVA followed by Bonferroni post hoc test. A *p* value less than 0.05 was considered significant.

## 3. Results

### 3.1. miR210 Knockout Provided Neuroprotection in Neonatal HI Brain Injury

**Figure 1:**
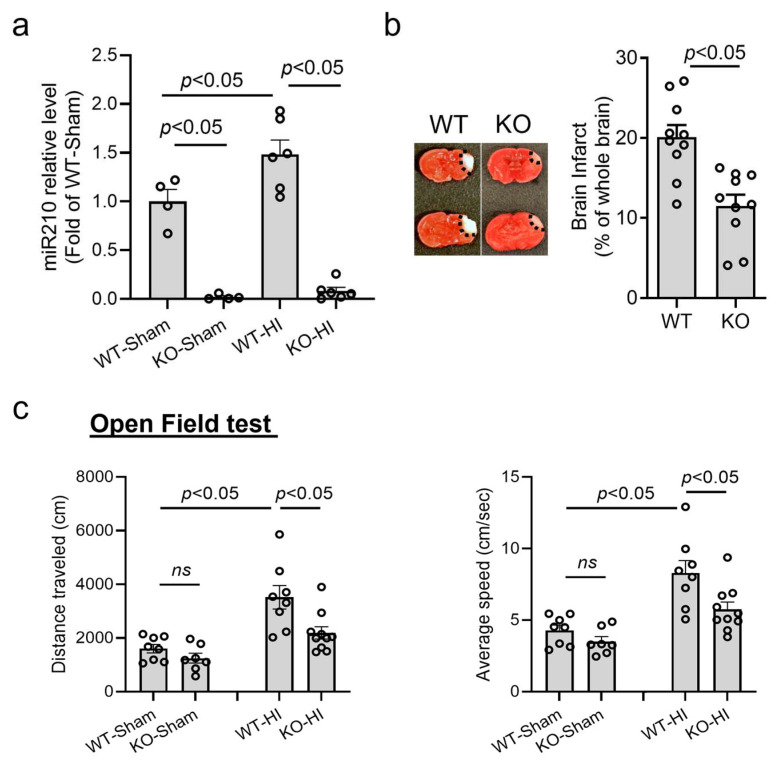
miR210 knockout (miR210 KO) provided neuroprotection in neonatal hypoxic–ischemic (HI) brain injury in mice. HI brain injury was conducted on postnatal day 9 (P9) miR210 KO or wild-type (WT) mouse pups. (**a**) RT-qPCR result of miR210 levels in the ipsilateral hemisphere of WT and miR210 KO pups at 24 h after HI. Data are presented as mean ± SEM. *n* = 4–6 pups per group. One-way ANOVA followed by Bonferroni post hoc test. (**b**) Representative images and quantitative result of brain infarct at 48 h after HI. Data are presented as mean ± SEM. *n* = 10 pups per group. Student’s *t* test (unpaired, two-tailed). (**c**) Open field test for locomotor function evaluation performed three weeks after HI. Data are presented as mean ± SEM. *n* = 7–10 pups per group. One-way ANOVA followed by Bonferroni post hoc test. *ns*, not significant.

Our previous studies have demonstrated that neonatal HI insult time-dependently upregulated miR210 in the brain of rodent pups, and miR210 inhibition by LNA antisense oligonucleotides provided neuroprotective effects after HIE [25]. Thus, using miR210 KO mice, we further confirmed the effect of miR210 on neonatal HI brain injury. HI insult was conducted on postnatal day 9 (P9) miR210 KO and WT mouse pups. The RT-qPCR results showed that brain miR210 was completely deleted in KO mice, compared with WT, and HI insult did not induce the upregulation of brain miR210 in KO pups at 24 h (Figure 1a). Moreover, miR210 KO significantly reduced brain infarct size (Figure 1b) at 48 h after HI, compared with WT (Figure 1b). To evaluate the effect of miR210 knockout on motor coordination function, an open field test was performed on one-month-old mice after HI brain injury (Figure 1c). The results revealed that neonatal HI insult induced a hyperactive state in WT mouse pups, showing increased average speed and travel distance, which was significantly reduced in miR210 KO pups. There was no significant difference observed between miR210 KO and WT pups in the sham groups.

### 3.2. miR210 Knockout Reduced Microglial Activation and Neuroinflammation After Neonatal HIE

**Figure 2:**
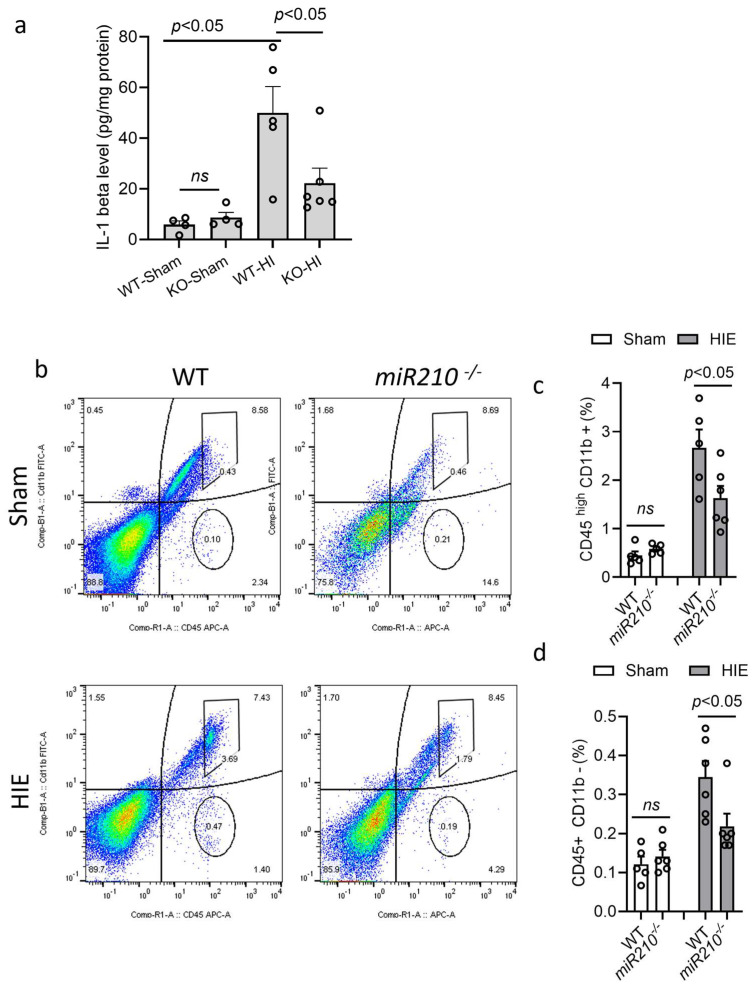
miR210 knockout reduced neuroinflammation in neonatal hypoxic–ischemic (HI) brain injury. (**a**) ELISA of IL1β levels in the ipsilateral hemisphere of WT and miR210 KO pups 24 h after HI insult. Data are presented as mean ± SEM. *n* = 4–6 pups/group. One-way ANOVA followed by Bonferroni post hoc test. (**b**–**d**) Representative images and quantitative result of flow cytometry of the proportion of activated microglia–macrophages (CD45 high/CD11b+) (**b**,**c**) and infiltrated lymphocytes (CD45+/CD11b-) (**b**,**d**) in the ipsilateral hemisphere of WT and miR210 KO pups 24 h after sham or HI insult. Colors show different particle density (high, red/orange; low, blue/green). Data are presented as mean ± SEM. *n* = 5–6 pups/group. One-way ANOVA followed by Bonferroni post hoc test. *ns*, not significant.

Wild-type (WT) or miR210 KO mice at postnatal day 9 (P9) were subjected to sham or HI insult. ELISA was conducted to measure the level of IL1β in the ipsilateral hemispheres after HIE. The result showed that HI insult significantly induced IL1β levels at 24 h in WT pups after HIE, compared with the sham, which was significantly reduced in KO mice. There was no significant difference in the level of IL1β between WT and KO mice in sham group (Figure 2a). Next, flow cytometry was performed to measure immune cell response after HI insult. The result showed that KO pups exhibited significantly reduced proportion of activated microglia–macrophage (CD45high/CD11b+) (Figure 2b,c) and lymphocytes (CD45+/CD11b-) (Figure 2b,d) in the ipsilateral hemisphere 24 h after HI, compared with WT. There was no significant difference observed between WT and KO mice in sham groups. These data indicate that miR210 knockout reduces microglia activation and neuroinflammatory response after neonatal HI brain injury.

### 3.3. miR210-LNA Inhibited the Expression of Proinflammatory Cytokine and Preserved Mitochondrial Respiratory Chain-Related Genes in Microglia After OGD

**Figure 3:**
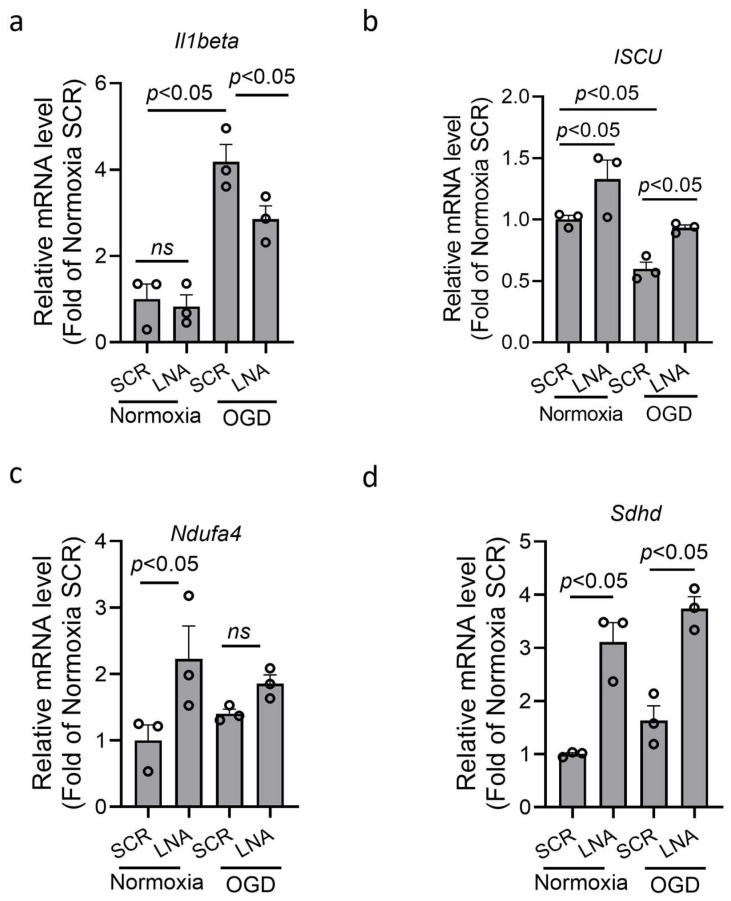
miR210 inhibition reduced the expression of proinflammatory cytokine and preserved the expression of mitochondrial OXPHOS-related genes in BV2 microglia after oxygen–glucose deprivation (OGD). BV2 cells were transfected with either miR210-LNA or LNA scramble (SCR) overnight followed by OGD treatment for 3 h. The RT-qPCR was performed for the transcript levels of *Il1b* (**a**), *ISCU* (**b**), *Ndufa4* (**c**), and *Sdhd* (**d**). Data are presented as mean ± SEM. *n* = 3 independent cell culture. One-way ANOVA followed by Bonferroni post hoc test. *ns*, not significant.

BV2 microglia were transfected with miR210 LNA inhibitor or scramble LNA (SCR) overnight followed by oxygen–glucose deprivation (OGD) or normoxia for 3 h. The result showed that OGD treatment significantly increased the mRNA level of proinflammatory cytokine IL1β in BV2 microglia, which was significantly reduced by LNA treatment (Figure 3a). MiR210 has been reported to negatively regulate genes encoding subunits of mitochondrial respiratory chain, including *ISCU*, *Ndufa4*, and *Sdhd* [25,26,39,40,41,42]. The RT-qPCR results showed that the expression of these genes was significantly upregulated after LNA treatment under normoxia. Under OGD, only *Iscu* was significantly reduced, compared with normoxia, which was rescued by LNA treatment (Figure 3b). The expression of *Ndufa4* and *Sdhd* was not significantly reduced by OGD compared with normoxia (Figure 3c,d).

### 3.4. miR210-LNA Preserved Mitochondrial OXPHOS and Weakened Glycolysis in Microglia After OGD

**Figure 4:**
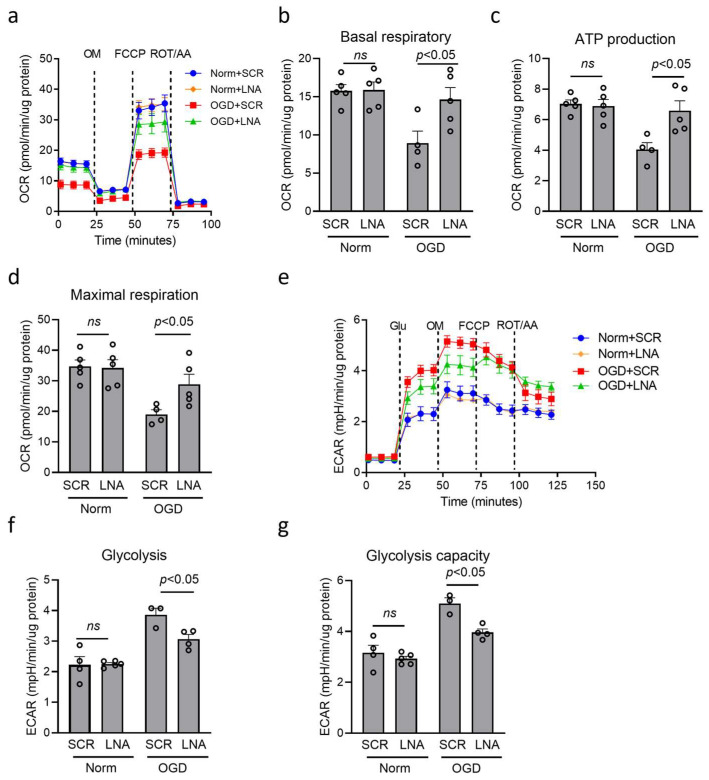
miR210 inhibition preserved mitochondrial OXPHOS and weakened glycolysis in BV2 microglia after OGD. BV2 cells were transfected with either miR210-LNA or LNA scramble (SCR) overnight followed by OGD treatment for 3 h. (**a**,**e**) Extracellular flux assay profiles including oxygen consumption rate (OCR) (**a**) and extracellular acidification rate (ECAR) (**e**), measured using the Agilent Seahorse analyzer. (**b**–**d**) OCR changes in basal respiratory (**b**), ATP production (**c**), and maximal respiration (**d**). (**f**,**g**) ECAR changes in basal glycolysis (**f**) and glycolysis capacity (**g**). Data are presented as mean ± SEM. *n* = 3–5 independent cell culture. One-way ANOVA followed by Bonferroni post hoc test. *ns*, not significant.

The metabolic change is a prerequisite for inflammatory regulation in macrophage/microglia [37,43,44]. Then, we detected the effect of miR210 on mitochondrial metabolism changes using a Seahorse XFe24 analyzer to measure oxygen consumption rate (OCR) (Figure 4a) and extracellular acidification rate (ECAR) (Figure 4e) under OGD. Our results showed that OGD treatment dampened OCR value, showing reduced basal respiratory (Figure 4b), ATP production (Figure 4c), and maximal respiration capacity (Figure 4d), which were significantly reversed by miR210-LNA treatment, compared with SCR. In addition, OGD treatment increased ECAR value, showing enhanced basal glycolysis (Figure 4f) and maximal glycolysis capacity (Figure 4g), which was significantly countered by miR210-LNA treatment, compared with SCR.

### 3.5. miR210 Regulated Proinflammation and Mitochondrial Gene Expression in Primary Microglia

**Figure 5:**
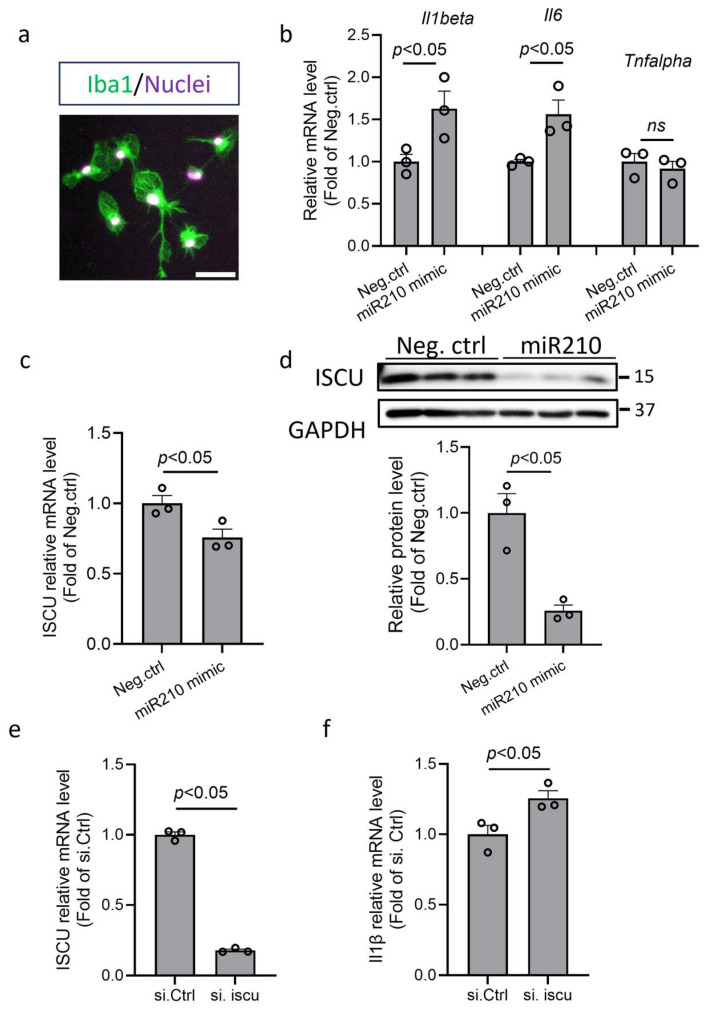
miR210 mimic upregulated proinflammatory cytokines and inhibited ISCU expression in microglia. (**a**) Representative image of isolated primary murine microglia stained with microglia marker Iba-1 (green). Scale bar: 50 µm. Primary microglia were transfected with miR210 mimic or control miRNA (Neg. Ctrl) for 48 h. Student’s *t* test (unpaired, two-tailed). (**b**,**c**) RT-qPCR results of the transcript levels of *Il1b*, *Il6*, *Tnf-α* (**b**), and *ISCU* (**c**). Data are presented as mean ± SEM. *n* = 3 independent cell culture. Student’s *t* test (unpaired, two-tailed). (**d**) Western blot result of the expression of ISCU. Data are presented as mean ± SEM. *n* = 3 independent cell culture. Student’s *t* test (unpaired, two-tailed). The blots were transferred to the PVDF from the same gel and cropped between 25 and 37 kDa for different primary antibody probing. Primary microglia were transfected with *ISCU* silencing RNA or control siRNA (Neg. Ctrl) for 48 h. (**e**,**f**) RT-qPCR results of the transcript levels of *ISCU* (**e**) and *Il1b* (**f**). Data are presented as mean ± SEM. *n* = 3 independent cell culture. Student’s *t* test (unpaired, two-tailed). *ns*, not significant.

To validate the role of miR210 in the proinflammatory response of microglia, primary murine microglia were isolated (Iba1-positive cells by immunofluorescence staining; Figure 5a) and transfected with miR210 mimic or negative control (Neg. Ctrl) for 48 h. The RT-qPCR results showed that miR210 mimic significantly increased mRNA levels of IL1β and IL6, but not TNFα (Figure 5b). Moreover, miR210 mimic treatment significantly reduced both mRNA (Figure 5c) and protein (Figure 5d) abundance of the ISCU gene. Our previous study has demonstrated the direct suppressive effect of miR210 on ISCU in neuronal cells [25]. The same as miR210 mimic, ISCU silencing RNA significantly reduced the transcript level of ISCU (Figure 5e) and upregulated the transcript level of IL1β (Figure 5f) in microglia, suggesting that miR210 and ISCU play a similar role in regulating microglia activation.

## 4. Discussion

In the present study, using miR210 knockout mice, we found that miR210 deletion provided neuroprotective effect after neonatal HI brain injury. Moreover, miR210 KO mouse pups exhibited significantly reduced production of proinflammatory cytokine IL1β and microglia activation after neonatal HIE. To test the mechanistic link between miR210 and microglial activation, we measured mitochondrial metabolic states and observed a shift from mitochondrial OXPHOS toward glycolysis after OGD. Moreover, miR210 inhibition preserved the balance between OXPHOS and glycolysis and reduced *Il1b* expression in microglial cultures, which may be attributed to the rescue of respiratory chain-related genes. In primary microglia, we found that miR210 mimic upregulated the expression of proinflammatory cytokines, which was similar to the effect observed with respiratory chain-related gene *ISCU* knockdown. Our results indicate that increased miR210 promotes microglial proinflammatory response through the disruption of mitochondrial bioenergetic processes under hypoxic–ischemic conditions.

Microglia are considered as one of the earliest responder cells to brain insults, and the major mediator of neuroinflammation [6,9]. During HIE, there is a diffuse activation of microglia in the neonatal brain. Once acutely activated, microglia secrete proinflammatory cytokines such as IL1β, TNFα, IL6, etc., and facilitate BBB opening and immune cell infiltration, leading to amplified neuroinflammation and further delayed neuronal death. The detrimental effects of proinflammatory activation of microglia, at least in the early phase of neonatal HI brain injury, have been well documented. And inhibition of microglial activation is considered as an efficient therapeutic approach for HIE treatment [45,46,47,48]. Our previous studies showed that miR210 inhibition by LNA reduced microglia activation and neuroinflammation, and provided neuroprotective effects on HI brain injury in rodent models of neonates and adults [24,48]. In the present study, using miR210 knockout mice and microglia culture, we further demonstrated that miR210 regulated the expression of proinflammatory cytokines in microglia after neonatal HI brain injury.

The mechanisms by which miR210 regulates microglial activation during HI remain elusive. It has been demonstrated that mitochondrial metabolic switch to glycolysis plays a key role in programming microglia–macrophage proinflammatory phenotype in the pathologies of many diseases [49,50,51,52,53]. Mitochondrial bioenergetic alteration is the very early event and a hallmark of neonatal HI brain injury [54,55]. Previous studies have demonstrated that miR210 decreases OXPHOS by repressing the expression of electron transport chain (ETC)-related genes, including *ISCU*, *Ndufa4*, *Sdhd* (complex I and II), and *Cox10* (complex IV) in multiple cell types such as neurons and endothelial cells. Our previous study has validated the direct suppressive effect of miR210 on *ISCU* in neuronal cells [25]. Consequently, the inhibition of the activity of ETC subunits, especially ISCU, leads to mitochondrial dysfunction and cell death [25,26,39,40,41,42]. In line with these studies, the present study showed that miR210 inhibition preserved OXPHOS and reduced the overactivation of glycolysis in BV2 microglia after OGD treatment. We also measured the expression of ETC-related genes, and found that *ISCU*, *Ndufa4*, and *Sdhd* were regulated by miR210 under normal conditions, whereas only *ISCU* was sensitive to HI in microglia. Therefore, our experiments focused on the effect of miR210-ISCU axis on microglial activation under HI conditions. Our results confirmed that not only in neuronal cells [25], miR210-ISCU axis also plays a key role in regulating mitochondrial functions in microglia after neonatal HI brain injury. The ISCU downregulation triggers the metabolic switch from oxidative phosphorylation to glycolysis, which promotes the inflammatory phenotypes in immune and non-immune cells through multiple molecular pathways. It can activate the Nuclear Factor of Activated T-cells (NFATs), the important transcription factors in regulating immune response [56]. It also regulates endothelial inflammation phenotype through the activation of Nuclear Factor kappa B (NFκB) and Nuclear Factor Erythroid 2-Related Actor 2 (NRF2) pathways, which mediate a positive feedback loop between glycolysis and inflammation [57]. In microglia, the glycolytic switch increases the expression of advanced glycation end products (AGEs), which bind to AGE receptors and stimulate the expression of proinflammatory genes [58,59]. In addition, our results also suggest that besides miR210, a complex regulatory mechanism exists for the expression of *Ndufa4* and *Sdhd* under HI. Mitochondria are the primary source of cellular reactive oxygen species (ROS) and are highly involved in oxidative stress. The disruption of ETC contributes to mitochondrial ROS overproduction, which is involved in regulating the microglia proinflammatory phenotype [60]. Our previous study determined that miR210 induced mitochondrial ROS production by the inhibition of ETC in neurons after HIE [25], and miR210 is the key regulator of mitochondrial ROS production in different tissues [61,62]. Moreover, activated microglia produce substantial ROS [63]. Therefore, oxidative stress and ROS overproduction in microglia would be the downstream effector of miR210 on microglial proinflammatory activation after hypoxia–ischemia. Compared to the in vitro studies, the regulation of miR210 on microglial activation in neonatal HIE could be more complicated. Our previous study demonstrated that miR210 inhibition reduced neuronal ROS and cell death after HIE [25]. As injured neurons release damage-associated molecular patterns (DAMPs), such as ATP and high-mobility group box 1 (HMGB1), resident immune cells such as microglia are activated in the brain, triggering the initiation of inflammatory cascade [64]. Therefore, in addition to the direct regulation of microglia activation, we cannot rule out the possibility that reduced microglia activation and neuroinflammation in miR210 KO pups after HIE may be attributed to the direct attenuation of neuronal injury.

## 5. Conclusions

In conclusion, we found that miR210 knockout reduced neuroinflammation after neonatal HI, and miR210 regulated microglial proinflammatory activation through regulating mitochondrial bioenergetic processes after hypoxia–ischemia in microglia. Our findings indicate that the effect of miR210 on mitochondrial metabolism by targeting ETC is highly conserved in multiple brain cell types. Our previous studies demonstrated the neuroprotective effect of miR210 inhibition in neonatal HI brain injury [25]. The present study provides new insight into the role of miR210 in regulating microglial proinflammatory phenotype in HIE, and adds new evidence to confirm that targeting miR210 is a potential therapeutic approach for neonatal HIE treatment.

## Data Availability

The original contributions presented in this study are included in the article. Further inquiries can be directed to the corresponding author.

## Abbreviations

CCA	Common carotid artery
CNS	Central nervous system
ELISA	Enzyme-linked immunosorbent assay
ETC	Electron transport chain
HIE	Hypoxic–ischemic encephalopathy
ISCU	Iron–sulfur cluster assembly enzyme
LNA	Locked nucleic acid
*miR210^-/-^*	miR210 knockout
OGD	Oxygen–glucose deprivation
OXPHOS	Oxidative phosphorylation
ROS	Reactive oxygen species
